# Phytochrome B1-dependent control of *SP5G* transcription is the basis of the night break and red to far-red light ratio effects in tomato flowering

**DOI:** 10.1186/s12870-018-1380-8

**Published:** 2018-08-06

**Authors:** Kai Cao, Fei Yan, Dawei Xu, Kaiqi Ai, Jie Yu, Encai Bao, Zhirong Zou

**Affiliations:** 1 The Agriculture Ministry Key Laboratory of Agricultural Engineering in the Middle and Lower Reaches of Yangze River, Jiangsu Academy of Agricultural Sciences, Nanjing, China; 2 Shaanxi Key Laboratory Bio-resources, Shaanxi University of Technology, Hanzhong, Shaanxi China; 3 Horticulture College, Northwest A&F University, Yangling, Shaanxi China; 4 Guangxi Zhong Nong Fu Yu International Agricultural Science and Technology Co., Ltd, Yulin, Guangxi China

**Keywords:** Tomato, Red to far-red light ratio, Night break, Phytochrome B1, Flowering, *SP5G*

## Abstract

**Background:**

Phytochromes are dimeric proteins with critical roles in perceiving day length and the environmental signals that trigger flowering. Night break (NB) and the red to far-red light ratio (R:FR) have been used extensively as tools to study the photoperiodic control of flowering. However, at the molecular level, little is known about the effect of NB and different R:FR values on flowering in day-neutral plants (DNPs) such as tomato.

**Results:**

Here, we show that tomato *SP5G*, *SP5G2*, and *SP5G3* are homologs of *Arabidopsis thaliana FLOWERING LOCUS T* (*FT*) that repress flowering in *Nicotiana benthamiana*. NB every 2 h at intensities of 10 μmol m^− 2^ s^− 1^ or lower R:FR (e.g., 0.6) caused a clear delay in tomato flowering and promoted *SP5G* mRNA expression. The promoted *SP5G* mRNA expression induced by red light NB and low R:FR treatments was reversed by a subsequent FR light stimulus or a higher R:FR treatment. The tomato *phyB1* mutation abolished the effects of NB and lower R:FR treatments on flowering and *SP5G* mRNA expression, indicating that the effects were mediated by phytochrome B1 in tomato.

**Conclusion:**

Our results strongly suggest that *SP5G* mRNA suppression is the principal cause of NB and lower R:FR effects on flowering in tomato.

**Electronic supplementary material:**

The online version of this article (10.1186/s12870-018-1380-8) contains supplementary material, which is available to authorized users.

## Background

Plants are sessile organisms that cannot therefore migrate from a suboptimal environment to a more favorable one. Thus, they have developed mechanisms that allow them to alter their growth and development in response to environmental signals, thereby increasing their likelihood of survival and reproductive success. The transition from the vegetative phase to the reproductive phase is a particularly well-studied example of how the external environment regulates plant development. Photoperiod is one of the most important regulators of flowering for successful reproduction [1]. Flowering plants can be divided into three groups according to their responses to photoperiod: long-day plants (LDPs), which flower faster under long-day (LD) conditions; short-day plants (SDPs), which are stimulated to flower under short-day (SD) conditions; and day-neutral plants (DNPs), which flower in a manner that is insensitive to the photoperiod.

In the LDP *Arabidopsis thaliana*, the expression of the *CONSTANS (CO)* is regulated by the circadian clock and subsequently induces *FLOWERING LCOUS T (FT)* expression when exposed to LD conditions [2, 3]. The genome of the SDP rice contains *Heading date 1* (*Hd1*) and *Heading date 3a* (*Hd3a*), homologs of *Arabidopsis CO* and *FT* that play important roles in the regulation of flowering [4, 5]. *Hd1* actives *Hd3a* expression under inductive SD conditions, whereas *Hd1* suppresses *Hd3a* under non-inductive LD conditions. The DNP tomato expresses four FT-like proteins (SP3D, SP5G, SP5G2, SP5G3). SFT/SP3D is a floral activator, and its expression is not altered by photoperiod [6]. SP5G is a floral repressor, and it is expressed at higher levels under LD conditions relative to SD conditions, which promote tomato flowering slightly earlier under SD conditions [6]. FT-like proteins that influence plant flowering have also been identified in dicotyledonous plants such as poplar [7, 8], apple [9], sugar beet [10], pumpkin [11], sunflower [12], pea [13], soybean [14], and potato [15], as well as monocotyledonous plants such as rice [16], wheat [17] and maize [18].

The night break (NB) effect on flowering has been discovered in both LDPs [19] and SDPs [20]. In *Arabidopsis*, a 1-h exposure to light given every day in the middle of the night can result in early flowering [19]. The NB effect on flowering is most evident in SDPs, in which flowering is inhibited by a very short exposure of light during the night. In rice, a 10-min NB had clear effects on flowering when applied for various numbers of days [20]. Early studies in rice on the light quality required for NB indicated that red (R) light is most effectively induces this response.

Phytochrome B is the major photoreceptor used for NB, which causes delayed flowering by suppressing the expression of *Hd3a* [20, 21]. In *Pharbitis*, another SDP, NB suppressed the expression of *PnFT1* and *PnFT2*, which are orthologs of *Arabidopsis FT* that induce late flowering [22]. The effect of NB on the flowering of DNPs has been investigated in few plants such as Geranium [23]. In addition to the duration of the light period, light quality (wavelength) is another one of the most important ambient signal for plants flowering. Plants growing under a canopy experience lower red (R) to far-red (FR) ratios (R:FR) than plants growing in full sun, because leaves absorb more R light than FR light. Low R:FR values are perceived by the phytochrome family of proteins and induce a range of responses including stem and petiole elongation, hyponastic leaves, reduced branching, and early flowering [1, 24]. For many species, FR-enriched light is known to influence plant flowering, but the molecular details are unknown. In *Arabidopsis*, FR-enriched light can increase CO protein levels independent of transcription and promote the expression of *FT* [25, 26].

Plants are able to respond properly to environmental changes because they have evolved multiple photoreceptor systems that utilize phytochromes, cryptochromes, and phototropins, which perceive light signals over a broad range of wavelengths and intensities. Phytochromes mainly perceive R and FR light, while cryptochromes and phototropins recognize blue light and UV-A [27]. Phytochromes are photochromic proteins that exist as two photo-interconvertible isomeric forms: the red-light-absorbing form (Pr) and the far-red-light-absorbing form (Pfr) [28]. Upon excitation by R or FR light (producing a high or low R:FR value, respectively), phytochrome converts Pr into Pfr or vice versa [28]. Phytochromes exist predominantly in the Pfr form in daylight and Pr form overnight, as dictated by the process of dark recovery [29]. The conversion between Pr and Pfr is used to synchronize plant development to the light environment. When *phyB* mutations occurred in the LDPs *Arabidopsis* [30] and pea [31] as well as the SDPs sorghum [32] and rice [33], early flowering occurred, with decreased photoperiodic sensitivities. Accordingly, *phyB* delays flowering by suppressing the expression of *FT*-like genes in LDPs and SDPs [21, 34]. There are five phytochromes in tomato: phyA, phyB1, phyB2, phyE, and phyF [35]. PHYB1 is involved in many tomato physiological and biological processes, such as de-etiolation, hypocotyl hook unfolding, cotyledon expansion, hypocotyl elongation, anthocyanin accumulation, and flowering [6, 35]. However, the functions of these phytochromes in integrating environmental signals into tomato flowering still remains unclear and requires further investigation.

Four expressed FT-like proteins, SP3D, SP5G, SP5G2, and SP5G3, have been identified previously by our lab, and they play important roles in tomato flowering [6]. Overexpression of these genes in *Arabidopsis* revealed that SP3D is a floral promoter, while SP5G, SP5G2, and SP5G3 are floral repressors [6]. To further study their roles in tomato flowering under different light conditions (i.e., NB and different R:FR values), we initially established the conditions required for efficient NB and R:FR values to impact flowering in tomato. The expression of *FT*-like genes after NB treatment and different R:FR values have been investigated in wild type (WT) and phytochrome mutants. The results clearly show that the increased *SP5G* mRNA is the principal cause of the NB and R:FR ratio effects on late flowering in tomato. Phytochrome B1 transduces the NB and R:FR signals, thereby influencing flowering.

## Results

### Overexpression of SP5G, SP5G2, and SP5G3 delayed flowering in Nicotiana benthamiana

**Fig. 1:**
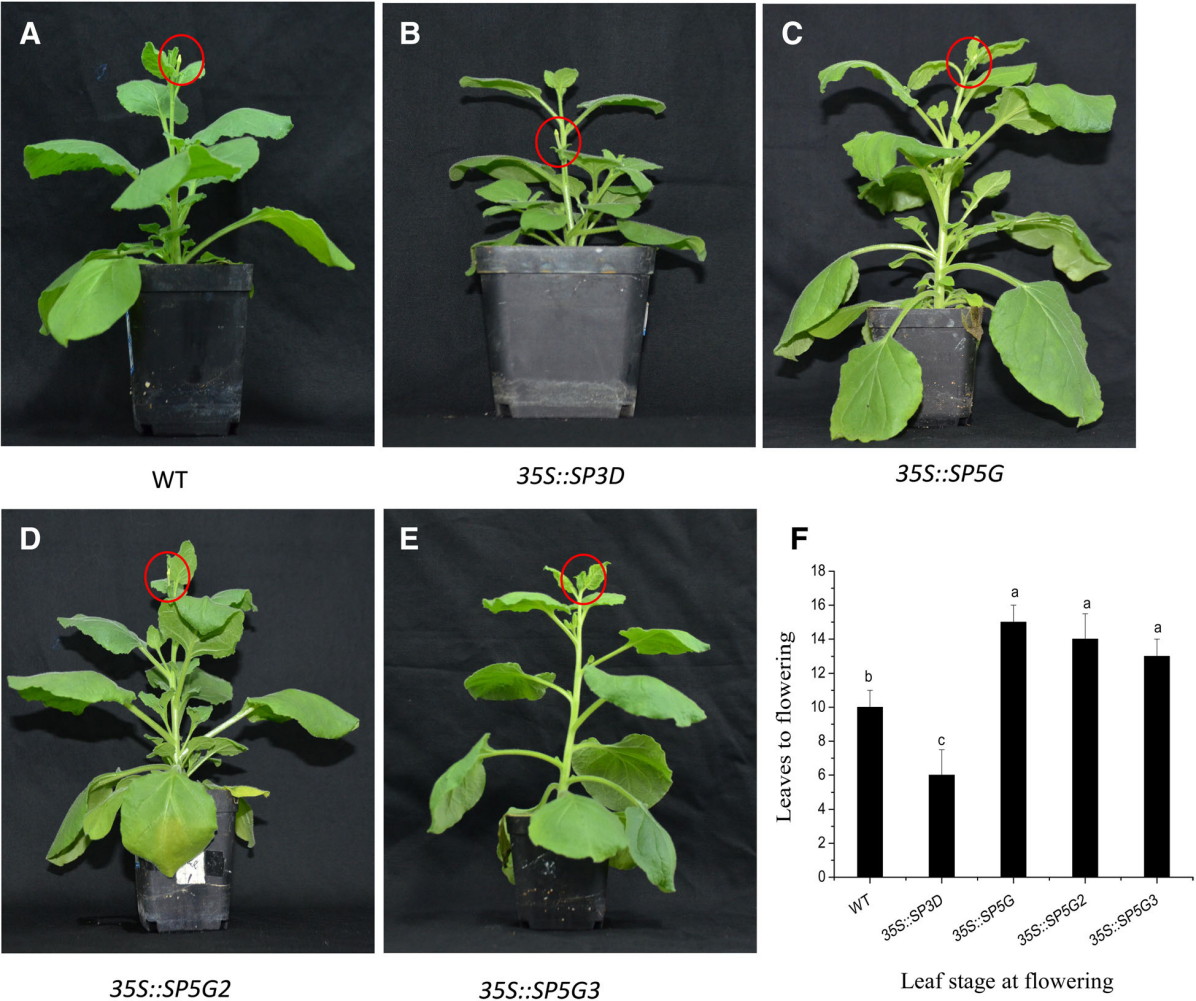
Overexpression of tomato *SP3D* promotes flowering, while overexpression of tomato *SP5G*, *SP5G2*, or *SP5G3* genes delay flowering in transgenic *Nicotiana benthamiana.* **a** WT, (**b**) *SP3D* overexpression line, (**c**) *SP5G* overexpression line, (**d**) *SP5G2* overexpression line, (**e**) *SP5G3* overexpression line, (**f**) leaf stage at flowering in *SP3D*, *SP5G*, *SP5G2*, and *SP5G3 Nicotiana benthamiana* overexpression lines under DN conditions. The red circles indicate flowers. Vertical bars represent the SE (*n* = 5). Bars with different letters are significantly different at the 0.05 level according to Duncan’s multiple range test

**Fig. 2:**
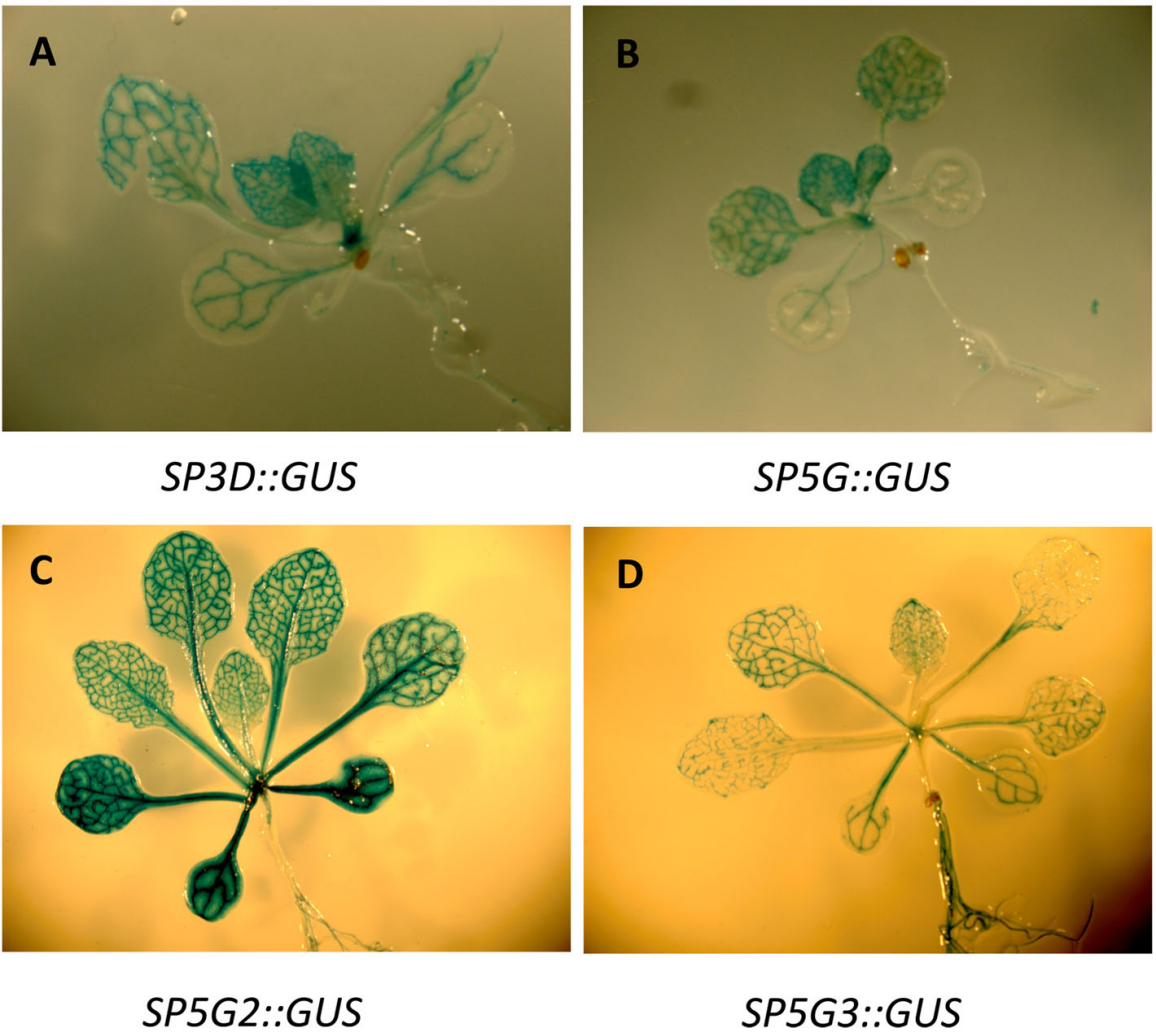
*SP3D-GUS*, *SP5G-GUS*, *SP5G2-GUS*, and *SP5G3-GUS* activity in transgenic *Arabidopsis thaliana*. GUS-activity was visualized with the chromogenic substrate X-Gluc. **a** *SP3D-GUS*, (**c**) *SP5G-GUS*, (**c**) *SP5G2-GUS*, and (**d**) *SP5G3-GUS*

We have previously reported that overexpression of *SP5G*, *SP5G2*, and *SP5G3* resulted in delayed flowering in transgenic *Arabidopsis* plants relative to WT controls [6]. To further investigate the functions of *SP3D*, *SP5G*, *SP5G2*, and *SP5G3* in flowering, we overexpressed tomato *SP3D*, *SP5G*, *SP5G2*, and *SP5G3* genes into *Nicotiana benthamiana.* Overexpression of *SP3D* leads to early flowering in transgenic *Nicotiana benthamiana* (Fig. 1a). However, overexpression of *SP5G*, *SP5G2*, or *SP5G3* delays flowering in transgenic *Nicotiana benthamiana* (Fig. 1b, c, d). Under day-neutral (DN) conditions, flowering occurs at the 10-leaf stage in WT plants. However, flowering occurred at the 6-leaf stage in *SP3D*-overexpressing plants, whereas flowering was delayed until the 15-leaf stage in *SP5G*-overexpressing plants, 14-leaf stage in *SP5G2*-overexpressing plants and 13-leaf stage in *SP5G3*-overexpressing plants under DN conditions (Fig. 1). These results indicate that *SP3D* promotes flowering, while *SP5G*, *SP5G2*, and *SP5G3* repress flowering. GUS (β-glucuronidase) staining can understand the organ, tissue and cell specificity of gene expression in transgenic plants. Transgenic *Arabidopsis* plants that carrying *SP3D-GUS*, *SP5G-GUS*, *SP5G2-GUS*, and *SP5G3-GUS* genes were stained to check GUS activity. All tested lines showed identical patterns of GUS staining, and differences were observed only in staining intensity. Histochemical examination of the transgenic *SP3D-GUS*, *SP5G-GUS*, *SP5G2-GUS*, and *SP5G3-GUS Arabidopsis* plants indicated that all transgenes were expressed in the vascular tissue of most of their organs (Fig. 2).

### Effects of NB and R:FR on flowering in tomato

**Fig. 3:**
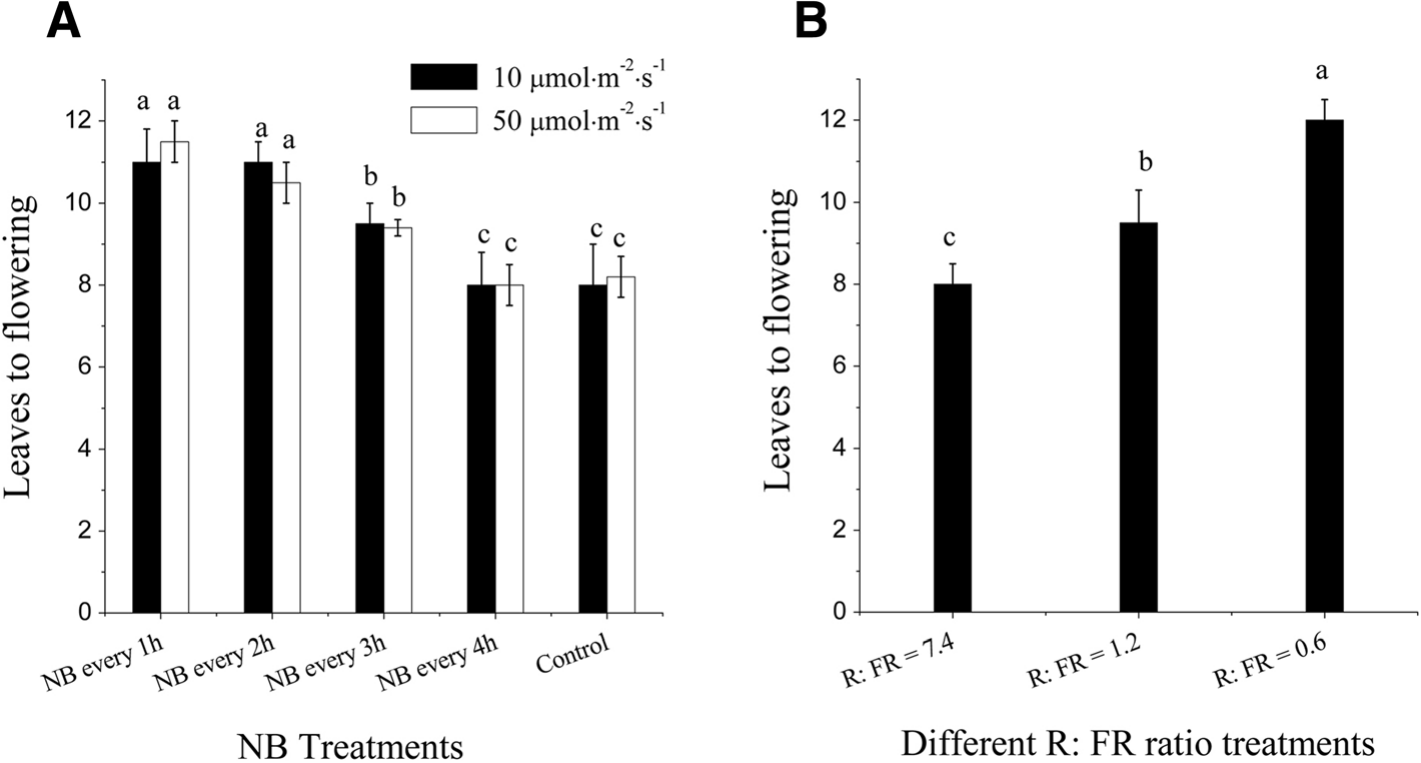
Effects of night break (NB) and different red-to-far-red light ratio (R:FR) treatments on the leaf stage at flowering in tomato plants. **a** Leaf stage at flowering under NB treatments every 1, 2, 3, or 4 h at one of two R light intensities, either 10 μmol m^− 2^ s^− 1^ or 50 μmol m^− 2^ s^− 1^. **b** Leaf stage at flowering for R:FR treatments, in which R:FR values are 7.4, 1.2, and 0.6. Vertical bars on the lines represent the SE (*n* = 10). Bars with different letters are significantly different at the *P* < 0.05 significance level according to Duncan’s multiple range test

To initiate a molecular-genetic study of the NB response in tomato, we first determined which NB frequencies and R light intensities elicited the most sensitivity to NB. We grew tomato seedlings under DN conditions, and the NB treatment was conducted from seed germination until flowering. Each burst of NB treatment lasted 10 min under various frequencies, which included NB every 1, 2, 3, or 4 h during the night at the intensities of 10 μmol m^− 2^ s^− 1^ and 50 μmol m^− 2^ s^− 1^. As a control, plants were raised with dark periods lacking NB. The leaf stage at flowering was recorded for both experimental and control plants. The NB effect on delayed flowering was detectable even when NB occurred every 3 h throughout the night (Fig. 3a). However, the strongest flowering delay effects occurred at the NB frequencies of 1 h and 2 h. NB at a light intensity of 10 μmol m^− 2^ s^− 1^ had a clear inhibitory effect on flowering in tomato. We next tested the effects of NB at a light intensity of 50 μmol m^− 2^ s^− 1^ on the inhibition of flowering. There was no clear difference in delay of flowering between the two light intensities (Fig. 3a).

For the R:FR experiments, tomato seedlings were grown under white LEDs alone or supplemented with FR LEDs. The R:FR ratios were 7.4, 1.2, and 0.6. A R:FR value of 0.6 is in the range of what plants might experience growing under a canopy [24]. In this study, we found that tomato seedlings grown under FR-enriched light conditions can exhibit delays in flowering while higher R:FR values (i.e., 7.4) lead to earlier flowering in tomato seedlings, relative to lower R: FR values (i.e., 0.6; Fig. 3b).

### *FT*-like gene expression under the NB and R:FR treatments

To examine the effects of NB and R:FR on gene expression, we assayed the expression of four *FT*-like genes that are important floral regulators in tomato. Previous studies have revealed that SP3D/SFT is a floral activator in tomato, while SP5G, SP5G2, and SP5G3 are floral repressors [6]. We measured the mRNA levels of the four *FT*-like genes by real time PCR over a 24-h period under DN conditions in the presence or absence of NB and different R:FR values.

**Fig. 4:**
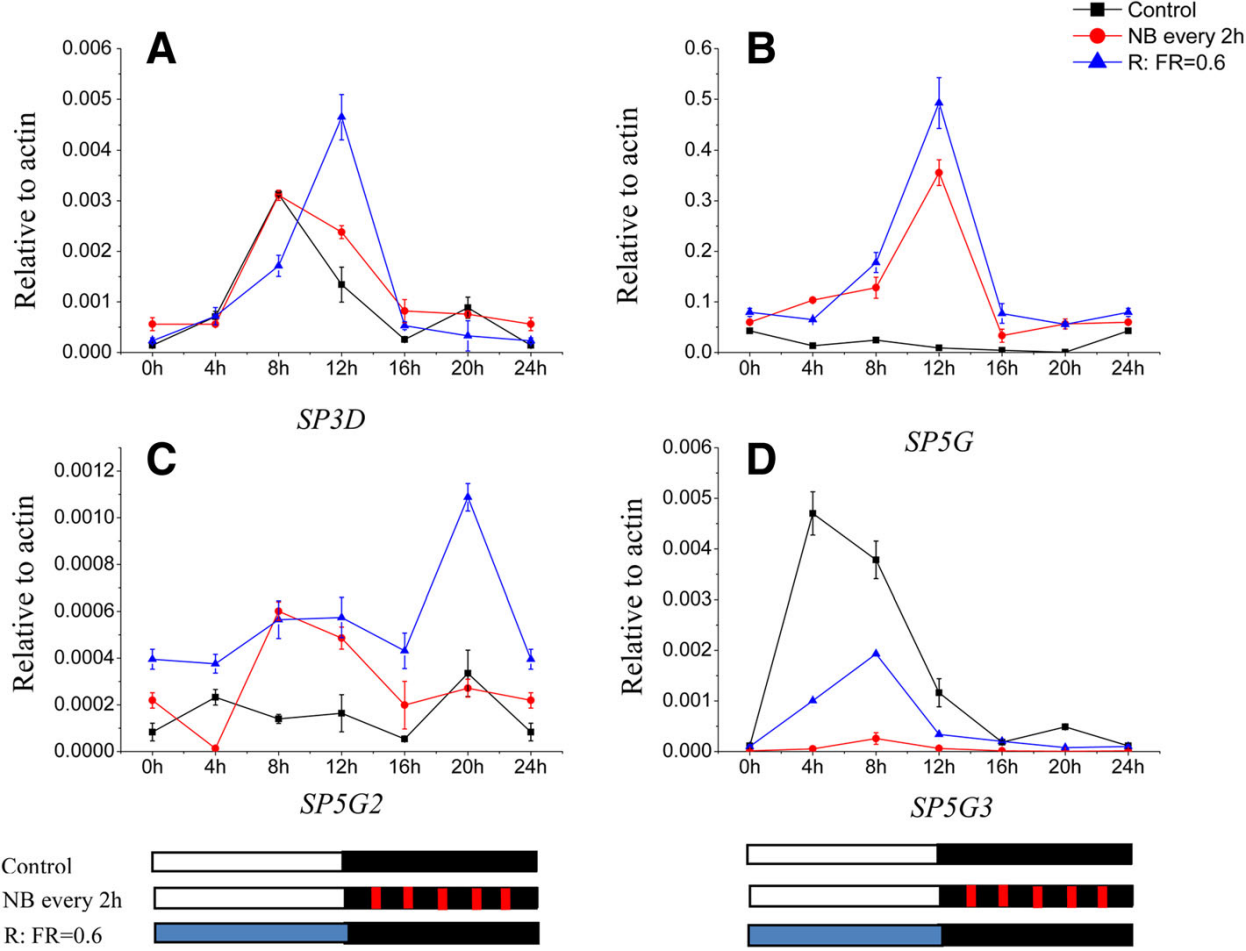
Diurnal expression of tomato *SP3D* (**a**), *SP5G* (**b**), *SP5G2* (**c**), and *SP5G3* (**d**) by night break (NB) and red-to-far-red light ratio (R:FR) treatments under day neutral (DN) conditions (12 h light/12 h dark), respectively. The black line represents the control (R:FR value is 7.4), the red line represents NB every 2 h, and the blue line represents a R:FR value of 0.6. Leaves were harvested from plants at 4-h intervals. The vertical axis shows relative mRNA levels of *FT*-like genes normalized to the expression of *Actin*. Error bars represent the standard error among technical replicates. White, black, red and blue bars at the bottom indicate light, dark red and far-red light periods, respectively

For the NB experiments, we applied 10 min of R light at a 2-h frequency throughout the 12-h night period. After the NB treatment, the expression of *SP5G* mRNA was strongly promoted during the day and night (Fig. 4b). Under normal conditions, *SP5G* mRNA was expressed at a very low level during the day and night. In contrast with *SP5G* mRNA, no clear effect was found on *SP3D* and *SP5G2* mRNA, and *SP5G3* mRNA showed the opposite pattern, i.e., a decrease after NB treatment (Fig. 4a, c, d). Therefore, the increased *SP5G* mRNA level likely delayed flowering in tomato plants under the NB treatment.

For the R:FR experiment, we supplied 12 h of supplemental FR light to produce R:FR values of 7.4 in the control and 0.6 in the treatment during the daytime. Under the FR-enriched light conditions, *SP5G* and *SP5G2* mRNA expression was strongly promoted during the day and night (Fig. 4b, c). However, *SP5G3* mRNA remained at a low level during the day and night under the lower R:FR value, and there was no significant influence on *SP3D* mRNA expression under the different R:FR values (Fig. 4a, d). Therefore, the increased *SP5G* and *SP5G2* mRNA levels may have delayed flowering in tomato plants under the lower R:FR value.

### The expression of *SP5G* mRNA can be reversed by R and FR light

**Fig. 5:**
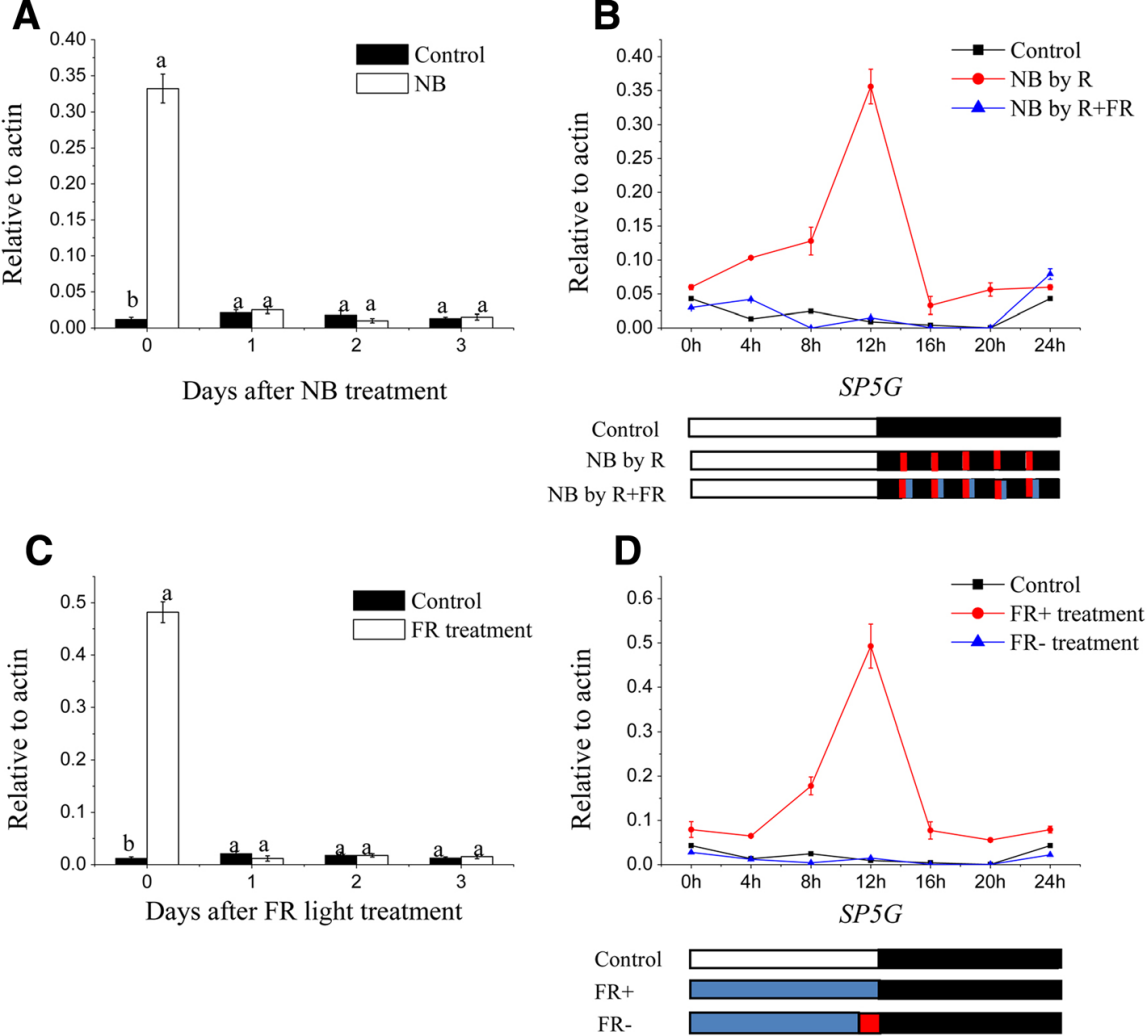
*SP5G* expression analysis conducted by qRT-PCR in tomato plants under different light treatments and DN conditions. **a** The expression of *SP5G* mRNA at 1, 2, and 3 days after the cessation of red (R) light night break (NB). **b** The diurnal expression of *SP5G* mRNA when tomato plants were treated by R light NB and red and far red (R + FR) light NB, respectively. **c** The expression of *SP5G* mRNA at 1, 2, and 3 days after cessation of far red (FR) light. **d** The diurnal expression of *SP5G* mRNA when tomato plants were treated with FR light and FR light was ended. All data are expressed as means ± SE of three independent pools of extracts. Three technical replicates were performed for each extract. Bars with different letters are significantly different at the *P* < 0.05 level according to Duncan’s multiple range test. White, black, red and blue bars at the bottom indicate light, dark red and far-red light periods, respectively

*SP5G* was recently shown to repress flowering, and it played a very important role in photoperiod response in tomato, so we focused on *SP5G* for further investigations [36]. To determine the length of the NB effect on *SP5G* mRNA expression, we examined *SP5G* mRNA at the end of light for 3 days without NB treatment after 2 weeks of R light NB treatments. The promotion of *SP5G* mRNA expression under the NB treatment completely disappeared by the next day after the NB treatment had been discontinued (Fig. 5a). To determine whether the increased expression of *SP5G* mRNA induced by R light NB can be reversed by FR light, we examined *SP5G* mRNA after exposure to 10 min of R light plus 10 min of FR light. *SP5G* mRNA was promoted by R light, and the NB effect was reversed by FR light (Fig. 5b). These results together clearly demonstrated that the upregulated *SP5G* mRNA expression is the basis of R light NB and that it influences tomato flowering.

To determine the duration of the FR light effect on *SP5G* mRNA expression after the cessation of FR light treatment, we examined *SP5G* mRNA in tomato plants grown under lower R:FR conditions for 2 weeks and then transferred them to higher R:FR light conditions for 3 days. The promotion of *SP5G* mRNA expression induced by lower R:FR values completely disappeared by the end of light in the following day after their transfer to higher R:FR conditions (Fig. 5c). To test whether the increased expression of *SP5G* mRNA in FR light-enriched condition can be reversed by R light, we examined *SP5G* mRNA expression after 10 min of FR light followed by cessation of the FR LED light. After the FR LED light was stopped, the expression of *SP5G* mRNA was almost at the same level as the control because white LEDs have a high R:FR ratio and phytochromes can convert Pr to Pfr under these conditions (Fig. 5d). The expression of *SP5G* mRNA was controlled by R:FR values in the day time and influenced tomato flowering.

### Phytochrome B1 is responsible for the effects of NB and R:FR on flowering and *SP5G* mRNA expression

**Fig. 6:**
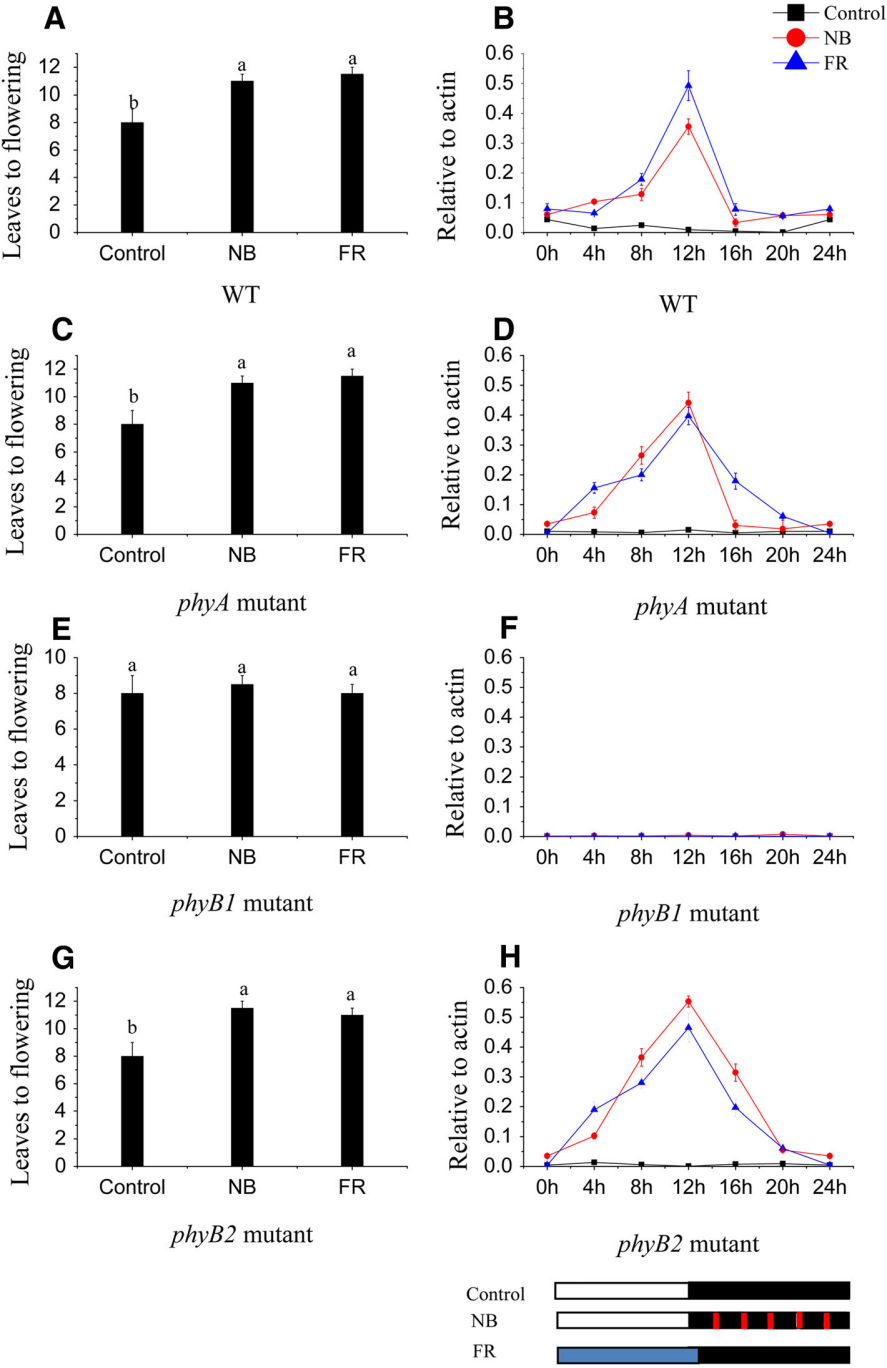
Phytochrome B1 is responsible for the expression of *SP5G* and influences the flowering of tomato plants by night break (NB) and different red-to-far-red light (R:FR) treatments. The leaf stage at flowering in tomato for (**a**) WT plants as well as (**c**) *phyA,* (**e**) *phyB1*, and (**g**) *phyB2* mutants treated by NB and different R:FR treatments. Data are mean ± SE of 10 plants. Bars with different letters are significantly different at the *P* < 0.05 significance level according to Duncan’s multiple range test. The diurnal expression of *SP5G* in tomato for (**b**) WT plants as well as (**d**) *phyA,* (**f**) *phyB1,* and (**h**) *phyB2* mutants treated by NB and different R: FR treatments. Data are expressed as means ± SE of three independent pools of extracts. Three technical replicates were performed for each extract. White, black, red and blue bars at the bottom indicate light, dark red and far-red light periods, respectively

Since the initial discovery of the NB effect on flowering, it has been well established that phytochrome is an important photoreceptor associated with the NB response [37]. The response to different R:FR values and shade is primarily regulated by phytochrome [38]. Therefore, we tested whether the NB and R:FR treatment effects on *SP5G* mRNA expression are mediated by phytochrome. Flowering time and *SP5G* mRNA expression were analyzed in WT plants as well as *phyA, phyB1*, and *phyB2* mutants after NB and under different R:FR treatments. The results on the effects of different phytochrome mutations on flowering under NB and different R:FR conditions clearly indicated that phyB1 is responsible for mediating the NB and different R:FR effects on the delay of flowering (Fig. 6e, f). The *phyA* and *phyB2* mutants flowered at leaf stages that were similar to those of the WT, while there were no effects on the flowering phenotype under the NB treatment and various R:FR treatments (Fig. 6a–d, g, h). Similarly, the NB and R:FR effects on the promotion of *SP5G* mRNA were abolished in *phyB1* mutants (Fig. 6f), whereas there was no clear, observable effect in *phyA* and *phyB2* mutants (Fig. 6d, h). Together, these results clearly demonstrated that phyB1 is responsible for delayed flowering and that *SP5G* mRNA expression was caused by the NB and R:FR treatments in tomato.

## Discussion

In the present study, overexpression of *SP5G*, *SP5G2*, or *SP5G3* in *Nicotiana benthamiana* delayed flowering relative to control plants (Fig. 1). Our previous phylogenetic analyses revealed that *SP5G*, *SP5G2*, and *SP5G3* are *FT*-like genes [6]. FT-like proteins form a sub-clade of phosphatidylethanolamine-binding proteins (PEBPs) and work as flower activators in many species [7–16]. Similar *FT*-like genes that function as floral repressors have been reported previously in *Beta vulgaris* (sugar beet) and *Nicotiana tabacum*. There are two *FT*-like genes in sugar beet, *BvFT1* and *BvFT2*, and they differ in three critical amino acid residues [10]. Tyr (134), Gly (137), and Trp (138) are the three most important amino acids BvFT2 proteins, and the modification of BvFT1 Asn (138) into Tyr, Gln (141) into Gly, and Gln (142) into Trp can completely revert its repressing function, thereby promoting flowering (Additional file 1: Figure S1) [10]. Amino acid sequence analysis revealed that tomato SP5G, SP5G2 and SP5G3 were not conserved, but SP3D are conserved in the three critical amino acid residues as observed in the BvFT2 protein (Additional file 1: Figure S1). The change of the ciritical amino acid may have resulted in SP5G, SP5G2, and SP5G3 becoming floral repressors (Additional file 1: Figure S1). One essential functional feature of *FT*-like genes is their expression in leaf vasculature and transport of the translated protein into the shoot apex. The expression of *SP5G, SP5G2* and *SP5G3* were detected mainly in vascular tissues (Fig. 2). According to the current model established in *Arabidopsis* and rice, *FT*-like genes are transcribed and translated in leaf vasculature and then move via the phloem to the shoot apical meristem, where they interact with an FD bZIP transcription factor to induce flowering [2, 3].

NB treatment and different R:FR values influence on plant flowering have been known for a long time [19, 21, 26]. Our results clearly demonstrate that NB every 2 h and lower R:FR values promote the subsequent accumulation of *SP5G* transcripts, resulting in a delay in tomato flowering (Figs. 3, 4). The effect of NB on flowering is most evident in SDPs, in which it inhibits *FT*-like gene expression, thus repressing flowering through a very short exposure of light during the night, especially by R light or high R:FR light [20, 22, 39]. In contrast, NB treatments promote flowering in LDPs, which consist of only a limited number of species that generally require longer light exposure times [19, 40]. Tomato is a DNP, and it accordingly flowers regardless of photoperiod, but flower initiation occurs earlier under SD conditions than LD conditions. In this study, when tomato seedlings are subjected to NB treatment, the leaf stage at flowering increased from 8 to 11 leaves, and *SP5G* mRNA expression significantly increased compared with the control. In nature, the values of R:FR can be used to sense proximity of neighboring plants or canopy vegetation, changes in day length, and seasonal variation, each of which influence flowering [24, 41]. Several studies have shown that the presence of FR light promotes flowering of LDPs, such as tussock bellflower (*Campanula carpatica*) and tickseed (*Coreopsis grandiflora*) [42], in which flowering was delayed when grown under photoselective filters that created an FR-deficient environment during the entire day. In contrast, the flowering of SDPs, such as strawberry [43], duckweed (*Lemna paucicostata*) [44] and chrysanthemum (*Chrysanthemum grandiflorum*) [45] were influenced by FR light environments. In this study, when tomato seedlings were subjected to lower R:FR treatments, the leaf stage at flowering increased from 8 to 11.5 leaves, and *SP5G* mRNA expression significantly increased compared with the control (Figs. 3, 4). Previous studies have reported similar findings of *FT*-like gene expression being influenced by different R:FR values [24–26].

Phytochromes are known to mainly perceive R and FR light, which influence plant growth and flowering in several crops [21, 46–48]. In chrysanthemum and soybean, flowering can be inhibited by R light NB treatment, which promotes the conversion of phytochrome to Pfr, thus inhibiting flowering. However, after a subsequent FR exposure, flowering inhibition imposed by R light could be reversed [49, 50]. Our results clearly demonstrated that the delayed flowering phenotype and increased SP5G mRNA expression induced by R light NB or lower R:FR treatment was reversed by subsequent FR light exposure or higher R:FR treatments (Fig. 5). These results indicated that phytochrome is involved in tomato flowering and *SP5G* mRNA expression by R light NB and different R:FR value treatments. In *Arabidopsis*, phytochrome B delays flowering by suppressing *FT* expression [34]. In rice, phyB is responsible for the delayed flowering and *Hd3a* mRNA suppression caused by NB treatments [21]. In this study, we found phyB1 is required for NB and lower R:FR value treatments to suppress flowering and promote *SP5G* mRNA expression, whereas *phyA* and *phyB2* have no effect on tomato flowering and *SP5G* mRNA expression via NB and various R:FR treatments. This suggests that phyB1 is the critical phytochrome that controls tomato flowering and *SP5G* mRNA expression (Fig. 6). Various light signal components have been shown to control CO stability throughout the day. FR light signals stabilize CO, R light signals destabilizes CO, and phyB is involved in CO degradation [47, 51]. Recent evidence has suggested that the function of PHYTOCHROME DEPENDENT LATE FLOWERING (PHL) counteracts the ability of phyB to regulate flowering, suggesting that the changes in CO stability are mediated by phyB [52]. Therefore, it is possible that the NB and different R:FR effects may be regulated by phyB by controlling the stability of the CO-like protein in tomato.

## Conclusion

In summary, we found *SP5G*, *SP5G2*, and *SP5G3* are FT-like genes, but overexpression of tomato *SP5G*, *SP5G2*, and *SP5G3* delays flowering in transgenic *Nicotiana benthamiana*. NB and lower R:FR treatments lead to delayed flowering phenotypes and increased *SP5G* mRNA expression in tomato, and phyB1 is required for this. We determined that *SP5G* is important for tomato flowering and that it is controlled by phyB1, which plays a very important role in integrating NB and R:FR signals. The present study provides a deeper understanding of the response of tomato flowering to different light conditions.

## Methods

### Plant material and growth conditions

The tomato variety MoneyMaker (*Solanum lycopersicum* L.) was used in this study. The MoneyMaker mutant backgrounds *phyA, phyB1*, and *phyB2* were provided by the Tomato Genetic Resource Center (Department of Vegetable Crops, University of California, Davis; TGR accession numbers LA4356, LA4357, and LA4358, respectively). Tomato seeds were soaked in 50% bleach for 30 min. After bleaching, seeds were rinsed thoroughly in running water, then sown directly onto moistened germination paper and incubated at 25 °C. After germination, seeds were sown into commercial substrate and grown in growth chambers for each of the different treatments.

### NB and FR treatments

**Fig. 7:**
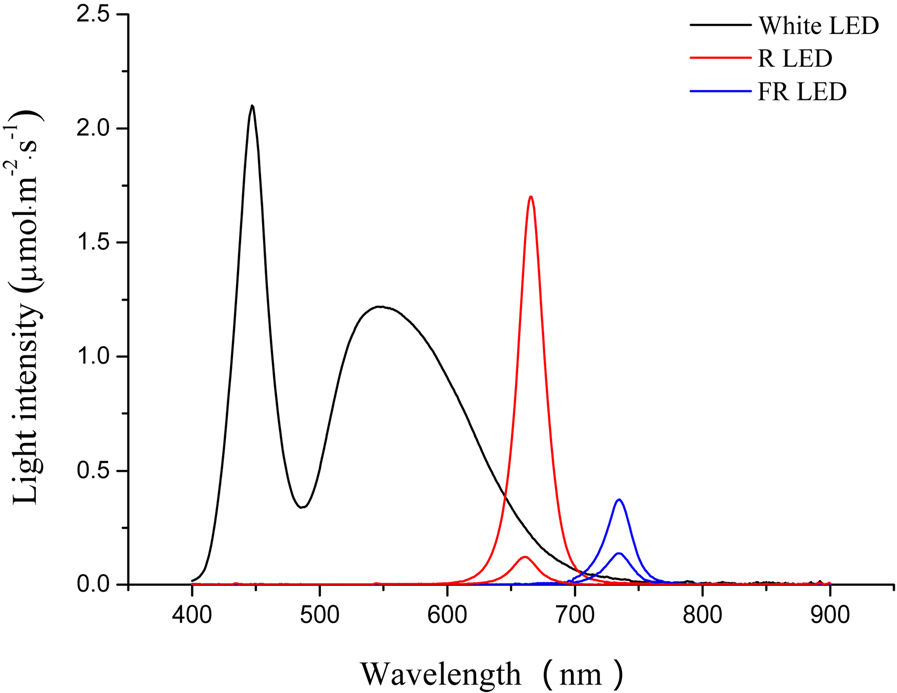
Spectral distribution characteristics of white, red (R) and far red (FR) LEDs used for night break (NB) and different R:FR treatments. The black curve represent white LED light; the two red curves represent two R light intensity, 10 and 50 μmol·m^− 2^·s^− 1^; and the two blue curves represent supplemental FR light to make R:FR values were1.2 and 0.6

For the NB studies, tomato seedlings were grown in growth chambers at 60% humidity under a photoperiod with daily cycles of 12 h of light at 25 °C and 12 h of darkness at 25 °C. Light was generated by an LED light source (400–700 nm, 200 μmol m^− 2^ s^− 1^). NB experiments were performed in the growth chambers using red LEDs (658 nm peak) with a light intensity of 10 μmol m^− 2^ s^− 1^ and 50 μmol m^− 2^ s^− 1^ at a NB stimulus delivered every 1, 2, 3, or 4 h throughout the night. For R:FR studies, tomato seedlings were grown at 25 °C with 60% humidity and under 200 μmol m^− 2^ s^− 1^ (400–700 nm) LED lights with or without supplemental far-red LEDs (730 nm peak), to yield R:FR values are 7.4, 1.2, and 0.6 as R:FR = (photon irradiance between 655 and 665 nm) / (photon irradiance between 725 and 735 nm). Light spectra were measured using a spectroradiometer (PAR-NIR; Apogee Instruments Inc., Logan, UT). The R light intensity and spectrum are shown in Fig. 7. The tomato seedlings were sub-irrigated every 3 days throughout the treatment with Yamasaki nutrient solution (pH 6.5 ± 0.1, electrical conductivity: 1.4–1.8 dS m^− 1^) containing 4 mmol/L NO_3_-N, 0.7 mmol/L NH_4_-N, 0.7 mmol/L P, 4 mmol/L K, 1.0 mmol/L Mg, 1.7 mmol/L Ca, and 2.7 mmol/L S as well as micro nutrients.

### RNA and DNA extraction

Tomato leaves were harvested at the end of the light, pooled from 3 third leaves of 5-week old plants, and total RNA was extracted using an RNeasy Plant mini kit (Takara, Dalian, China) following the manufacturer’s instructions. Plant genomic DNA was isolated using an Easy Pure Plant Genomic DNAkit (TransGen, Beijing, China) according to the manufacturer’s instructions.

### Gene isolation, vector construction, and plant transformation

The ORFs of *SP3D*(Solyc03g063100), *SP5G* (Solyc05g053850), *SP5G2* (Solyc11g008640), *SP5G3* (Solyc11g008650) were PCR amplified from the cDNA of MoneyMaker, cloned into a pENTR/3C vector (Invitrogen, Shanghai, China), and then subcloned into the pBCO-DC vector by recombination using the LR Clonase enzyme (Invitrogen, Shanghai, China). pBCO-DC carries a spectinomycin resistance gene for bacterial selection and a Basta resistance gene for selection of transformed plants. To express GUS under the control of tomato *SP3D*, *SP5G*, *SP5G2*, and *SP5G3* promoters, 3000 bp of 5′ upstream sequence of these genes was amplified from MoneyMaker genomic DNA, cloned into a pENTR/3C vector, and then transferred into a pK7WGF2 vector [53] by recombination using the LR Clonase enzyme. The bacterial resistance of pK7WGF2 is spectinomycin, and plant selectable marker is kanamycin. The primers used are listed in Additional file 1: Table S1. The plasmid mediated by *Agrobacterium tumefaciens* strain CV3101 was transformed into *Nicotiana benthamiana* and *Arabidopsis thaliana*. Transformed plants were selected on 0.8% agar media containing Murashige and Skoog salts, 0.5 g/L MES, and 10 g/L sucrose containing 10 μg ml^− 1^ basta or kanamycin. After screening for regenerated shoots on selection medium containing basta or kanamycin, the transgenic plants were further verified by PCR using genomic DNA as a template and 35S forward and gene-specific reverse primers.

### β-glucuronidase (GUS) activity assay

Seedlings were grown on selected MS media with Kana antibiotics until they reached the 3–4 true leaf stage. For the histochemical GUS assay, the seedlings were incubated in X-GLUC reaction buffer (2 mM X-GLUC [5-bromo-4-chloro-3-indolyl-β-d-glucuronide cyclohexylamine salt)] 1 mM EDTA, 50 mM NaPO_4_, 0.5 mM K_3_Fe(CN)_6_, 0.5 mM K_4_Fe(CN)_6_, and 1% Triton X-100) overnight at 37 °C. The seedlings were cleared by a series of ethanol extractions after an overnight reaction, and blue precipitates were examined under a dissecting microscope.

### Gene expression studies

For study the diurnal expression of *SP3D*, *SP5G*, *SP5G2*, and *SP5G3* genes, tomato leaves were harvested every 4 h for 24 h (0, 4, 8, 12, 16, 20, and 24 h), pooled from 3 third leaves of 5-week old plants. Total RNA was extracted using an RNeasy Plant mini kit (Takara, Dalian, China) following the manufacturer’s instructions. cDNA synthesis was performed by using the SuperscriptIII First strand synthesis system (Invitrogen, Shanghai, China) following the manufacturer’s instructions. Real-time PCR was performed using SYBR Premix Ex Taq (Takara, Dalian, China) in a Bio-Rad CFX96 real-time PCR system (Bio-Rad, Hercules, CA, USA). As an internal control gene, *actin* transcripts were assayed. The primers used are listed in Additional file 1: Table S1. Real-time quantitative PCR was repeated three times, and each time every sample was assayed in triplicate by PCR.

### Statistical analysis

Statistics were calculated using SPSS 20.0 (SPSS, version 20.0, IBM Inc., Armonk, NY, USA). The data were analyzed using an analysis of variance (ANOVA), and the differences between the means were assessed using Duncan’s multiple range test (*P* < 0.05). Error bars in all figures represent standard deviations from the mean. Graphs were created using OriginPro (version 8.0, Origin Lab, Northampton, MA, USA).

## Additional file

Additional file 1: **Table S1.** Sequences of primers used in this study for plasmid construction and quantitative RT-PCR. **Figure S1.** Partial amino acid alignment of tomato *FT*-like sequences and other PEBP family proteins. Vertical arrowheads indicate amino acids essential for AtFT activity (Tyr85/Gln140) versus AtTFL1 activity (His88/Asp144). The red shaded area is part of exon 4, which encodes an external loop that has evolved very rapidly among TFL1 homologs, but is almost invariant in FT homologs. The yellow shaded area indicates amino acids that are important for the antagonistic activities of *FT*-like genes in tomato and sugar beet. (PDF 197 kb)

## Abbreviations

DNPs: Day-neutral plants
FR: Far-red
GUS: β-glucuronidase
LDPs: Long day plants
NB: Night break
Pfr: Far-red-light-absorbing form
Pr: Red-light-absorbing form
R: Red
R:FR: Red to far-red light ratio
SDPs: Short day plants
