# Supplementary material for: Phytochrome B1-dependent control of SP5G transcription is the basis of the night break and red to far-red light ratio effects in tomato flowering
Source: BMC Plant Biol. 2018 Aug 6;18:158. doi: 10.1186/s12870-018-1380-8 (PMC6080379; doi:10.1186/s12870-018-1380-8)
Supplement: Supplementary file 1 — Table S1. Sequences of primers used in this study for plasmid construction and quantitative RT-PCR. Figure S1. Partial amino acid alignment of tomato FT-like sequences and other PEBP family proteins. Vertical arrowheads indicate amino acids essential for AtFT activity (Tyr85/Gln140) versus AtTFL1 activity (His88/Asp144). The red shaded area is part of exon 4, which encodes an external loop that has evolved very rapidly among TFL1 homologs, but is almost invariant in FT homologs. The yellow shaded area indicates amino acids that are important for the antagonistic activities of FT-like genes in tomato and sugar beet. (PDF 197 kb) [file 12870_2018_1380_MOESM1_ESM.pdf]

TITLE

Phytochrome B1-Dependent Quantitative Control of *SP5G* mRNA Expression is the Basis of the Night Break and Red to Far-Red Light Ratio Effects on Tomato Flowering

AUTHOR

Kai Cao[1, 2], Da-Wei Xu[1], Kai-Qi Ai[1], Jie Yu[1], En-Cai Bao[1] and Zhi-Rong Zou[1]*

Table S1. Sequences of primers used in this study for plasmid construction and quantitative RT-PCR.

Gene		Primers (5'-3')
**For plasmid construction**		
SP3D	F	CATCGTCGACAATGCCTAGAGAACGTGATCCTC
	R	AACTGCGGCCGCTCAATCAGCAGATCTTCTACGTC
SP5G	F	CATCGTCGACAATGCCTAGAGATCCTTTAATAGTTTCTG
	R	AACTGCGGCCGCTTATAGGCGACGACCACCGG
SP5G2	F	CATCGTCGACAATGCAAAGAGAAAGAGATACATTGAGAC
	R	AACTGCGGCCGCTTATATTCTACGACCACCAGTACCA
SP5G3	F	CATCGTCGACAATGAAGTTATATGTTATGAGTCCAAGGC
	R	AACTGCGGCCGCTTATTCGCAACGACGACCAC
SP3D promoter	F	CATCGTCGACATACGAATTTCTATGTTAGTG
	R	AACTGCGGCCGCGACGATGGTTGACGATAAACA
SP5G promoter	F	CATCGTCGACATCACAAAAAGTTTATTGAACAAAAC
	R	GCGCGCGGCCGCGATTTTTTTTCTTCTCAATTATAATTA
SP5G2 promoter	F	CATCGTCGACATAAAGAATCAAATCCTTTCCCAATAAAA
	R	AACTGCGGCCGCGATGAAATACTATTTAAGCAATGAGTCA
SP5G3 promoter	F	CATCGTCGACACCGAGTCGATCTATGAGGCC
	R	GCGCGCGGCCGCTCCCTATAATAATAATACAA
**For quantitative RT-PCR**		
SP3D	F	GTTGGTCGTGTGGTAGGGGA
	R	GCCTAAGCTCGCATCCATTA
SP5G	F	CTAGCAACCCAAACCTGAGG
	R	ATTGCCAAAGGTTGCTCCTG
SP5G2	F	GATTTGAGGCCTTCTATGGT
	R	ATCCACCATAACCAGAGTGT
SP5G3	F	TTATATGTTATGAGTCCAAGGC
	R	GTATTGAAATTTTGACGCCA
Actin	F	ATTCCCTGACTGTTTGCTAGT
	R	GCCATCTTATGCTATTCCTTTT

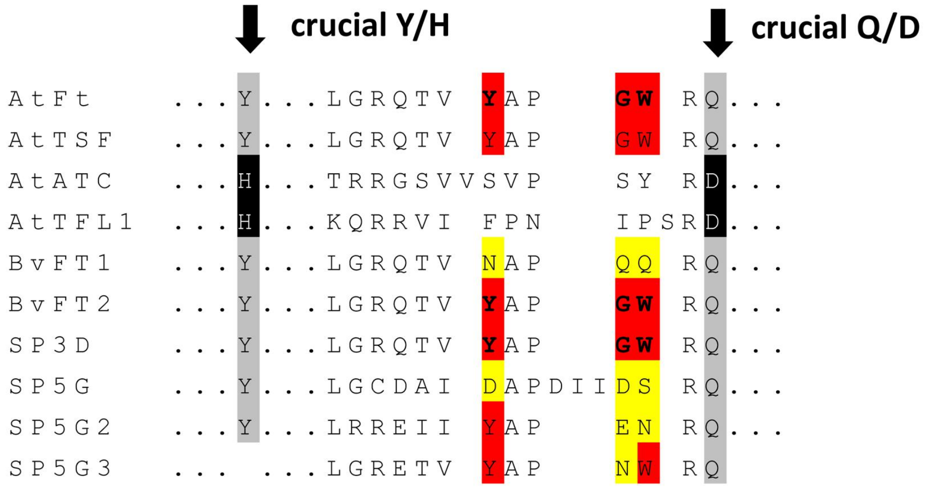

Figure S1. Partial amino acid alignment of tomato *FT*-like sequences and other PEBP family proteins. Vertical arrowheads indicate amino acids essential for AtFT activity (Tyr85/Gln140) versus AtTFL1 activity (His88/Asp144). The red shaded area is part of exon 4, which encodes an external loop that has evolved very rapidly among TFL1 homologs, but is almost invariant in FT homologs. The yellow shaded area indicates amino acids that are important for the antagonistic activities of *FT*-like genes in tomato and sugar beet.
